# Deep Insight into the *Ganoderma lucidum* by Comprehensive Analysis of Its Transcriptome

**DOI:** 10.1371/journal.pone.0044031

**Published:** 2012-08-27

**Authors:** Guo-Jun Yu, Man Wang, Jie Huang, Ya-Lin Yin, Yi-Jie Chen, Shuai Jiang, Yan-Xia Jin, Xian-Qing Lan, Barry Hon Cheung Wong, Yi Liang, Hui Sun

**Affiliations:** 1 State Key Laboratory of Virology, College of Life Sciences, Wuhan University, Wuhan, People’s Republic of China; 2 Department of Clinical Immunology, Guangdong Medical College, Dongguan, People’s Republic of China; 3 Key Laboratory of Fermentation Engineering (Ministry of Education), Hubei University of Technology, Wuhan, People’s Republic of China; 4 Key Laboratory of Combinatorial Biosynthesis and Drug Discovery (Ministry of Education), Wuhan University, Wuhan, People’s Republic of China; Cinvestav, Mexico

## Abstract

**Background:**

*Ganoderma lucidum* is a basidiomycete white rot fungus and is of medicinal importance in China, Japan and other countries in the Asiatic region. To date, much research has been performed in identifying the medicinal ingredients in *Ganoderma lucidum*. Despite its important therapeutic effects in disease, little is known about *Ganoderma lucidum* at the genomic level. In order to gain a molecular understanding of this fungus, we utilized Illumina high-throughput technology to sequence and analyze the transcriptome of *Ganoderma lucidum*.

**Methodology/Principal Findings:**

We obtained 6,439,690 and 6,416,670 high-quality reads from the mycelium and fruiting body of *Ganoderma lucidum*, and these were assembled to form 18,892 and 27,408 unigenes, respectively. A similarity search was performed against the NCBI non-redundant nucleotide database and a customized database composed of five fungal genomes. 11,098 and 8, 775 unigenes were matched to the NCBI non-redundant nucleotide database and our customized database, respectively. All unigenes were subjected to annotation by Gene Ontology, Eukaryotic Orthologous Group terms and Kyoto Encyclopedia of Genes and Genomes. Differentially expressed genes from the *Ganoderma lucidum* mycelium and fruiting body stage were analyzed, resulting in the identification of 13 unigenes which are involved in the terpenoid backbone biosynthesis pathway. Quantitative real-time PCR was used to confirm the expression levels of these unigenes. *Ganoderma lucidum* was also studied for wood degrading activity and a total of 22 putative FOLymes (fungal oxidative lignin enzymes) and 120 CAZymes (carbohydrate-active enzymes) were predicted from our *Ganoderma lucidum* transcriptome.

**Conclusions:**

Our study provides comprehensive gene expression information on *Ganoderma lucidum* at the transcriptional level, which will form the foundation for functional genomics studies in this fungus. The use of Illumina sequencing technology has made de novo transcriptome assembly and gene expression analysis possible in species that lack full genome information.

## Introduction

*Ganoderma lucidum* is a basidiomycete white rot fungi and has been used as traditional herbal medicine in Asia for thousands of years [1]. It has an abundance of bioactive components, which have numerous positive effects in diseases with little side effects [2]–[5]. Furthermore, this fungus has been used as a potent resource for lignocellulose degrading enzymes for many years [6]–[8].

The main pharmaceutical ingredients of *Ganoderma lucidum* include polysaccharides, triterpenoids, proteins, peptides, adenosine and nucleosides [9]–[12]. To date, most research on *Ganoderma lucidum* has focused on polysaccharides (GLPS) and ganoderic acids (GAs). Over 200 polysaccharides have been isolated from the fruiting bodies and mycelia of this fungus, among these, β-1–3 and β-1–6 D-glucans are the major bioactive components [10], [13], [14]. Their multiple pharmacological effects, such as immunomodulation, antioxidation, antitumor activity and especially hepatoprotection against chemical or immune hepatic damage, have been demonstrated in numerous animal models [15]–[18]. The class of triterpenes has received considerable attention because of their significant hepatoprotective effects [19], [20]. To date, over 130 triterpenes have been isolated from *G. lucidum* [21] and these compounds have been divided into the C30, C27 and C24 classes according to their number of carbon atoms, structure and functional groups [22], [23]. Except for these, LZ-8, a protein isolated from *G. lucidum* mycelia, has been shown to have immunomodulating activity [12], [24], and its structural, biochemical and immunological properties have been studied in much detail [25]–[28]. Moreover, numerous laccase isozymes which have ligninolytic capabilities have also been identified from *G. lucidum* [6], [7], [29].

In recent years, next-generation sequencing techniques (such as Roche 454, Illumina Solexa GA, and SOLiD) has helped to improve the efficiency of discovering novel genes and has provided a platform for further understanding differential gene expression at the genomic and transcriptional level [30]–[33]. While much attention has been given to human [34] and the model organisms [35]–[38], genome research of other non-model organisms such as macroscopic fungi remain in its infancy.

At present, only genomes from fungi with economical importance such as functions in biofuel production are available. These genomes have been studied in much detail and include: *Phanerochaete chrysosporium* [39]–[41], *Pichia stipitis* [42], *Trichoderma reesei* [43], *Laccaria bicolor* [44]–[46], *Postia placenta* [47], [48], *Coprinopsis cinerea* [49], *Schizophyllum commune* [50]. Considering these, sequence of transcriptome makes intensive study of organisms which lack genomes possible, such as *Cimex lectularius* [51], *Nilaparvata lugens* [52], *Bemisia tabaci* [53], *Dunaliella tertiolecta* [54], *Camellia sinensis* [55] and *Bactrocera dorsalis* [56]. Moreover, transcriptome could authentically reveal the expression level of some genes from an organism (such as fungi) living in a specific environment and provides great help for identifying interested genes.

Although *G. lucidum* has been extensively studied from a pharmacological perspective, genomic resource, molecular information on developmental processes and the content of lignocellulose degrading enzymes for *G. lucidum* are scarce. At present, only 1,046 expressed sequence tags (ESTs) of *G. lucidum* are obtainable from the NCBI public database [57]. While these sequences are a valuable resource for analysis in *G. lucidum*, the current genetic data is insufficient for elucidating the molecular mechanisms of *G. lucidum* from a pharmacological and developmental perspective.

In this study, we determined the transcriptomes of two developmental stages (mycelium and fruiting body) from *G. lucidum* using Illumina sequencing technology, for the purpose of further understanding the differential expression of genes relating to important (pharmaceutical) metabolic pathways and lignocelluloses degradation. In the current work, we obtained over 12 million high-quality 90 bp DNA fragments and demonstrated the efficiency and practicability of short-read sequencing for de novo assembly and annotation of genes expressed in a eukaryote without prior genome information. We identified 28,210 unigenes (18,892 unigenes in mycelium and 27,408 in fruiting body), and analyzed the gene expression profiles of *G. lucidum* at two different developmental stages by using a digital gene expression system. By transcriptome analysis, our results provide an important resource for identifying the effective pharmaceutical ingredients and new lignocelluloses degrading enzymes in *G. lucidum* and for analyzing the developmental process of this fungus.

## Results and Discussion

### Illumina Sequencing and De novo Assembly

To obtain an overview of the *G. lucidum* gene expression profiles during different developmental stages, cDNA samples were prepared from *G. lucidum* mycelia and fruiting bodies and sequenced using the Illumina HiSeq™ 2000 sequencing platform. After filtering for adaptor sequences, duplication sequences, ambiguous reads and low-quality reads, a total of over 12.8 million clean reads 90 bp long were obtained in a single sequencing run, of which 6,439,690 reads belonged to mycelium and 6,416,670 reads to the fruiting body (Table 1). Then, these high quality reads were assembled to produce 18,892 mycelium unigenes and 27,408 fruiting body unigenes using SOAP de novo and TGICL software. The mean sizes of mycelium and fruiting body unigenes were 498 bp and 514 bp, respectively, with lengths ranging from 100 bp to over 3,000 bp.

**Table 1:** General features of the *Ganoderma lucidum* transcriptome.

	Mycelium	Fruiting body	Total
**Number of reads**	6,439,690	6,416,670	12,856,360
**Total Nucleotides** **(bp)**	579,572,100	577,500,300	1,157,072,400
**Average read** **length (bp)**	90	90	90
**Number of contigs**	36,634	155,864	192,498
**Average contig** **length (bp)**	309	163	–
**Number of** **scaffolds**	26,276	44,308	70,584
**Average scaffold** **length (bp)**	410	371	–
**Number of** **unigenes**	18,892	27,408	28,210
**Length of all** **unigenes (bp)**	9,408,216	14,087,712	14,302,470
**Average unigene** **length (bp)**	498	514	507

**Figure 1:**
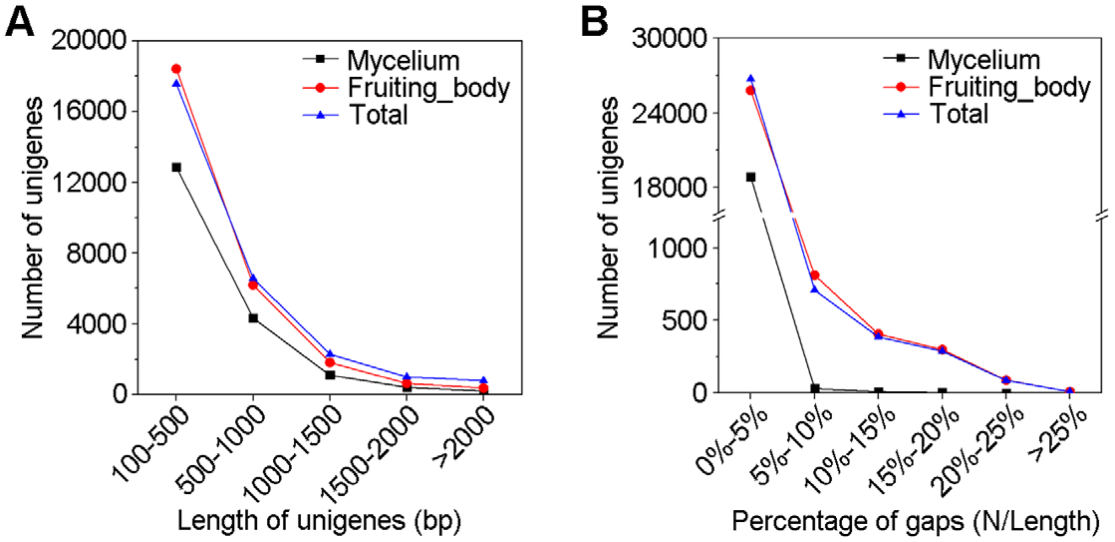
Overview of the assembled unigenes in *Ganoderma lucidum* transcriptome. (A) Length distribution of assembled unigenes. The length of unigenes ranged from 100 bp to over 2000 bp. (B) Gaps distribution of unigenes. Gaps represent the ratio of uncertain bases in unigenes. The majority of unigenes contained gaps ranging from 0% to 5%.

Unigenes from each sample were assembled together by sequence clustering software TGICL resulting in 28,210 all-unigenes. The length of the majority of unigenes was 100–500 bp, with 12,841 from mycelium, 18,400 from the fruiting body and 17,558 from the all-unigenes set (Figure 1A).

To evaluate the quality of the dataset, the ratio of the gap length of assembled unigenes was assessed (Figure 1B). The majority of the unigenes showed gap lengths less than 5% of the total length, which accounted for 99.8% of mycelium unigenes, 94.1% of fruiting body unigenes and 94.8% of all-unigenes. Few unigenes had a 30% or greater gap, indicating that the assembled data was of high quality.

### Functional Annotation of *G. lucidum* Transcriptome

**Figure 2:**
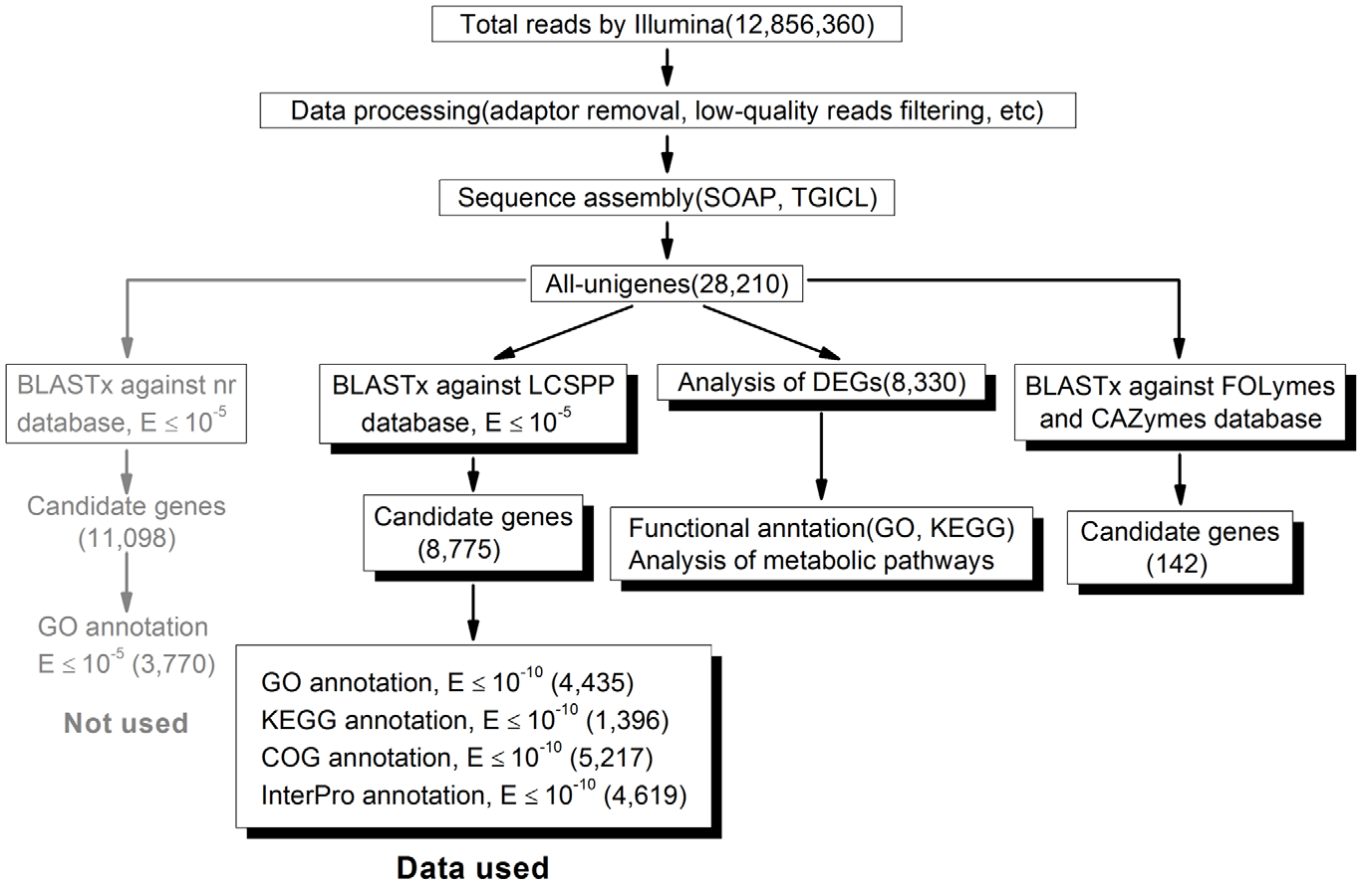
Analysis pipeline of *de novo* transcriptome assembly and annotation of *G. lucidum*.

An outline flow of the transcriptome analysis procedure is shown in Figure 2. For annotation, all unigenes were aligned against sequences from the National Center for Biotechnology Information (NCBI) non-redundant (nr) protein database by using the BLASTx algorithm with an E-value threshold of 10^−5^. 11,098 out of 28,210 unigenes (39.3%) were found to match to known proteins (Figure 2). Most sequences in the nr database were from non-fungal organisms and other fungal species other than *Basidiomycota*. *G. lucidum* is member of *Agaricomycotina* in *Basidiomycota*. While 39.3% of all-unigenes matched to the nr database, only 3,770 of 11,098 unigenes received GO annotation.

To analyze the *G. lucidum* transcriptome in further detail, a customized fungal database (LCSPP database) consisting of genome sequences of *Laccaria bicolor*, *Coprinopsis cinerea*, *Schizophyllum commune*, *Phanerochaete chrysosporium*, *Postia placenta*, which all belonged to the *Basidiomycota* family, was constructed. These fungal genomes are available on the U.S. Department of Energy Joint Genome Institute website [58]. BLASTx search was performed against this LCSPP database with an E-value cutoff of 10^−5^. 8,775 unigenes (31.1%) were found to match to the LCSPP database (Figure 2).

The proportion distribution of sequences matching to different fungi species is shown in Figure S1. The *G. lucidum* transcriptome produced a strong match against the *Schizophyllum commune* genome. To further understand the *G. lucidum* transcriptome, genomic information (13,181 genes) of *S. commune* [50] was downloaded from the JGI website and subjected to the same functional annotation as *G. lucidum*.

#### 1) Gene ontology annotation

**Figure 3:**
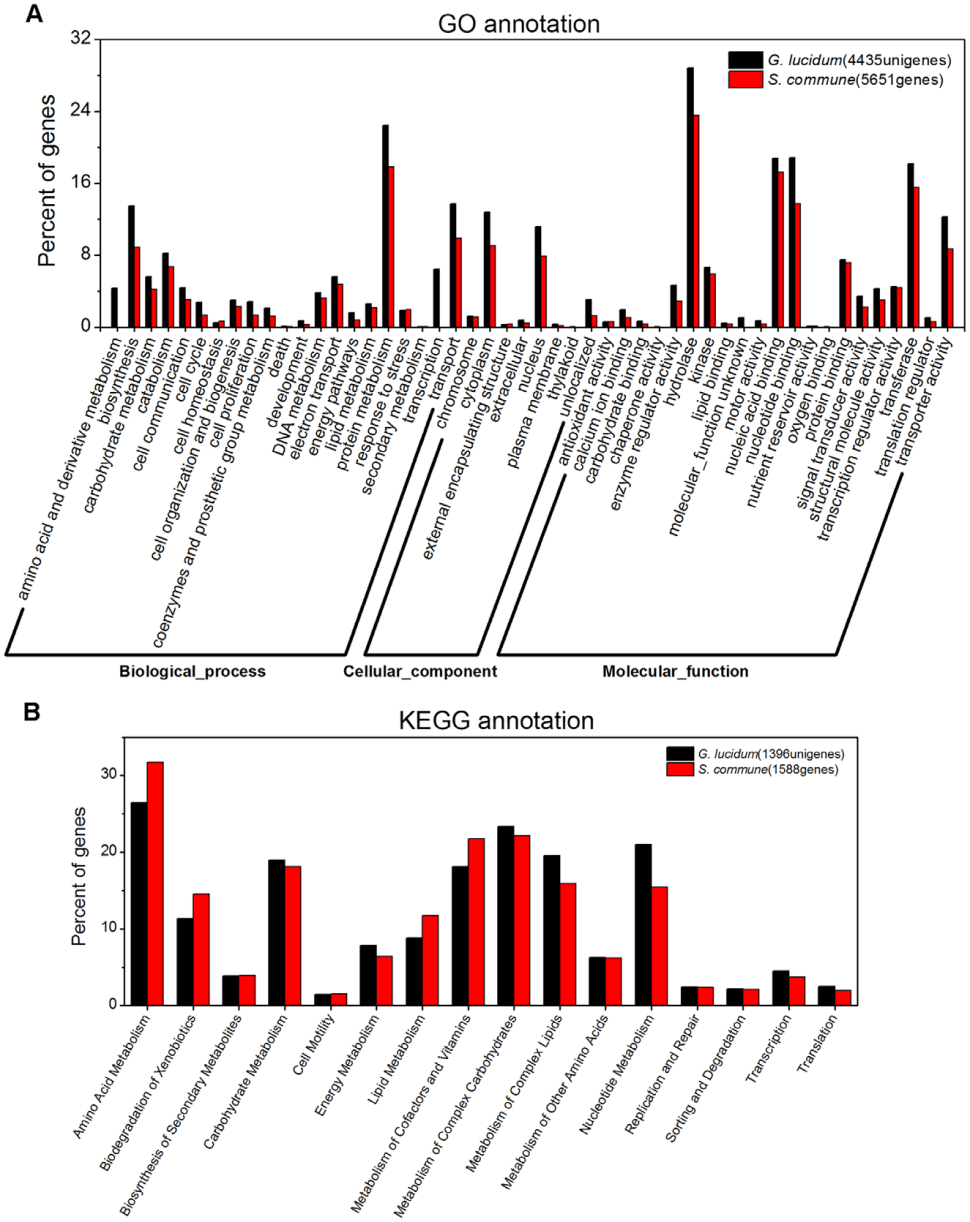
Annotation of the *G. lucidum* transcriptome by Gene Ontology and KEGG classification. (A) GO annotation. Gene Ontology classification is summarized into three main categories: biological process, cellular component and molecular function. 4,435 unigenes from *G. lucidum* and 5,651 genes from *S. commune* were subjected to GO annotation. The x-axis represents the GO annotation categories and the y-axis represents the percentage of genes of a specific category/total matched unigenes. Some genes have more than one GO annotation. (B) 1,396 unigenes (*G. lucidum*) and 1,588 genes (*S. commune*) were subjected to KEGG annotation and were grouped into 16 metabolic pathway classes.

Based on a sequence homology search against the LCSPP database using an E-value cutoff of 10^−10^, 4,435 unigenes were annotated across the GO sub-categories (Table S1). These unigenes were grouped into 50 functional groups with 21 involved in biological process, 8 in cellular component and 21 in molecular function. 5,651 genes of *S. commune* got GO annotation, and were also grouped into the 50 functional groups. As shown in Figure 3A, the *G. lucidum* and *S. commune* revealed similar trends in GO annotation. Among these GO classifications, “biosynthesis” (13.48%, 598), “protein metabolism” (22.46%, 996), “transport” (13.73%, 609), “hydrolase” (28.86%, 1280), “nucleic acid binding” (18.76%, 832) and “transferase” (18.17%, 806) were dominant in *G. lucidum*. However, few genes relating to “death”, “secondary metabolism”, “chaperone activity”, “nutrient reservoir activity” and “oxygen binding” were found.

#### 2) KEGG annotation

KEGG metabolic pathways analysis was performed by initially aligning unigenes with sequences from the LCSPP database and automatically assigning gene functions to the corresponding KEGG terms. 1,396 annotated unigenes from the *G. lucidum* transcriptome and 1,588 genes of *S. commune* were grouped into 16 known metabolic or signaling pathway classes (Table S2). As illustrated in Figure 3B, *G* *. lucidum* and *S. commune* produced similar trends in distribution in KEGG annotation. The pathways containing most unigenes in *G. lucidum* were involved in “Amino Acid Metabolism” (26.43%), “Carbohydrate Metabolism” (18.98%), “Metabolism of Cofactors and Vitamins” (23.35%) and “Metabolism of Complex Carbohydrates” (23.35%), which are all involved in the maintenance of basic biological processes of *G. lucidum*.

#### 3) COG annotation and protein domains in *G. lucidum* transcriptome

To further evaluate the effectiveness of our annotation process and the completeness of our transcriptome, we searched the annotated sequences for the genes involved in COG classifications. A total of 5,217 unigenes were assigned to one or more COG functional categories using BLASTx with an E-value threshold of 10^−10^. 7,160 genes of *S. commune* got COG annotation. Out of the 25 COG categories (Figure S2), “General function prediction only” was the most populated group (15.99%) followed by “Signal transduction mechanisms” (11.27%) and “Posttranslational modification, protein turnover, chaperones” (10.83%). The least populated groups were: “Cell motility” (0.19%), “Cell wall/membrane/envelope biogenesis” (1.32%) and “Extracellular structures” (1.4%).

A homology search for conserved protein domains was performed against the InterPro database using an E-value cutoff of 10^−10^. 4,619 unigenes were annotated, in which AAA ATPase (IPR003593, 265, 5.74%) was the most dominant protein domain, followed by protein kinase (IPR000719, 236, 5.11%), G-protein beta WD-40 repeat (IPR001680, 181, 3.92%), and helicase, C-terminal (IPR001650, 171, 3.70%) (Table S3). These annotations provide a valuable resource for further investigating the *Ganoderma lucidum*.

### Analysis of Differentially Expressed Genes

**Figure 4:**
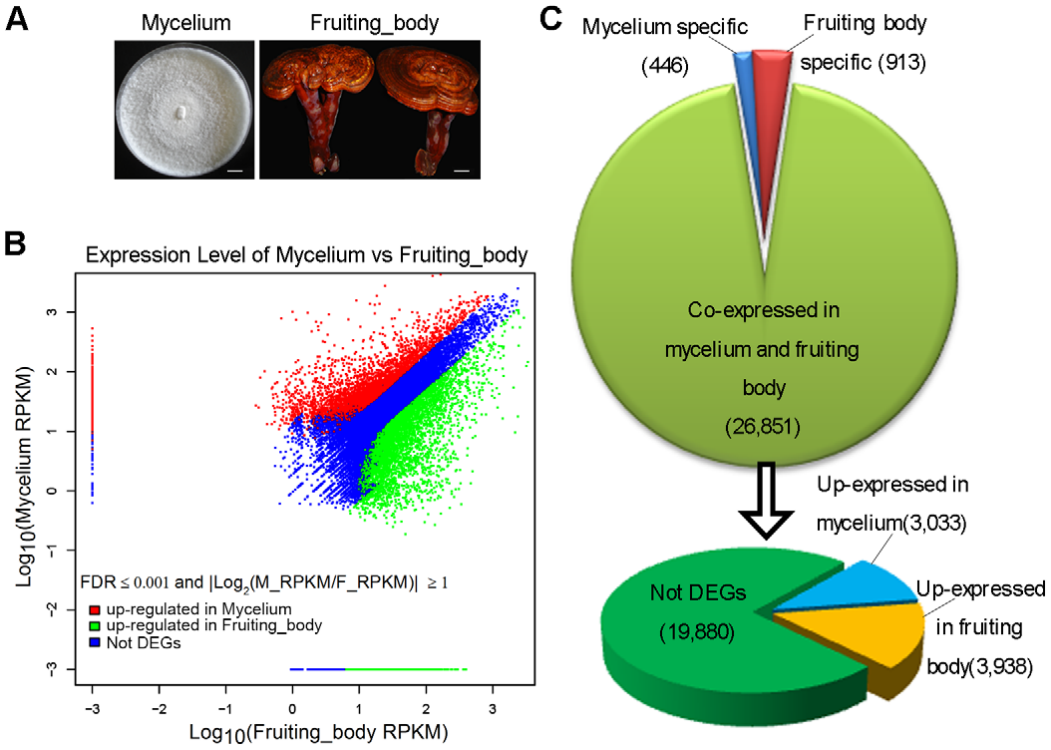
Summary of differentially expressed genes (DEGs) between *G. lucidum* mycelium and fruiting body. (A) Representative pictures of a vegetative mycelium from *G. lucidum* at 16 days (left) and a fruiting body at 80 days (right), scale bar = 1 cm. (B) Scatter plot of total unigenes from the *G. lucidum* transcriptome. The scatter plot shows the expression levels of 28,210 unigenes. The data was normalized as RPKM values and represented on a log_10_ scale. Red areas represent unigenes that showed up- regulated expression in mycelium, green areas represent unigenes that had up-regulated expression in the fruiting body and the blue areas represent unigenes showing no significant expression difference between the two developmental stages. (C) Overview of DEGs between *G. lucidum* mycelium and fruiting body. Out of a total of 28,210 unigenes, 446 unigenes were mycelium specific and 913 unigenes were fruiting body specific. Among the 26,851 co-expressed unigenes, 3,033 unigenes were up-regulated in mycelium and 3,938 unigenes were up-regulated in the fruiting body.

To identify differentially expressed genes (DEGs) between the *G. lucidum* mycelium and fruiting body (Figure 4A), the expression level for each unigene was calculated using the RPKM method [59] (Figure 4B). Out of 28,210 unigenes, 8,330 unigenes were differentially expressed between *G. lucidum* mycelium and fruiting body, of which 913 genes were specifically expressed in fruiting body and 446 in mycelium. 3,938 and 3,033 unigenes showed up-regulated expression in fruiting body and mycelium, respectively (Figure 4C, Table S4). 19,880 unigenes did not show significant expression changes between the two development stages.

#### 1) KEGG annotation analysis of DEGs

**Figure 5:**
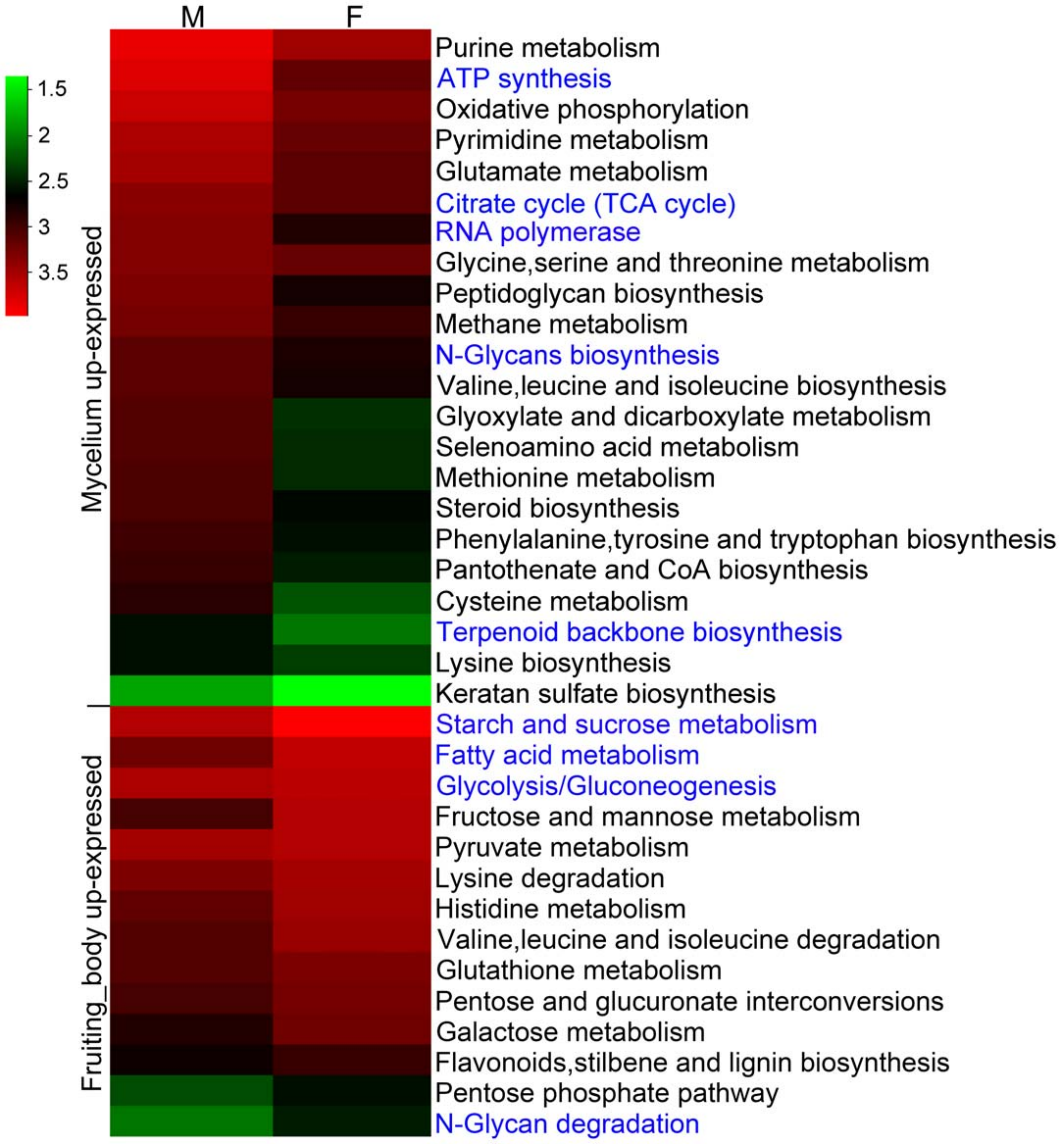
KEGG annotation of DEGs. The heatmap shows 36 of 100 annotated pathways of DEGs between *G. lucidum* mycelium (M) and fruiting body (F). Among the 36 pathways, 22 pathways were up- regulated in mycelium, and the rest of the pathways showed up-regulated expression in the fruiting body. Different colors represent different expression level of a particular metabolic pathway during the two development stages. Green color represents down-regulated expression and red color represents up-regulated expression. Each row represents a differentially expressed metabolic pathway. The data used to construct this heatmap was based on the log_10_ value of the RPKM values of all unigenes relating to a particular metabolic pathway in mycelium or fruiting body stage.

To study the function of DEGs, functional annotation was adopted for these identified genes. The metabolic and regulatory pathways relating to these DEGs were analyzed by using the log (base 10) value of the RPKM values (Figure 5). Metabolic pathways relating to biosynthesis were over-expressed at the transcriptional level in the mycelium stage whereas pathways mainly involved in degradation were more active in the fruiting body. For example, “ATP synthesis”, “Citrate cycle (TCA cycle)”, “RNA polymerase” and “N-Glycans biosynthesis” showed high transcriptional activity at the mycelium stage, whereas “Starch and sucrose metabolism”, “Fatty acid metabolism”, “Glycolysis/Gluconeogenesis” and “N-Glycan degradation” were more active during the fruiting body stage. These expression differences are indicative of their importance at their respective developmental stages. Furthermore, the pathways of “Purine metabolism”, “Starch and sucrose metabolism”, “Glycolysis/Gluconeogenesis”, “Pyruvate metabolism”, were highly expressed in both developmental stages, demonstrating the importance of these pathways during *G. lucidum* development.

#### 2) Analysis of GO enrichment in DEGs

**Figure 6:**
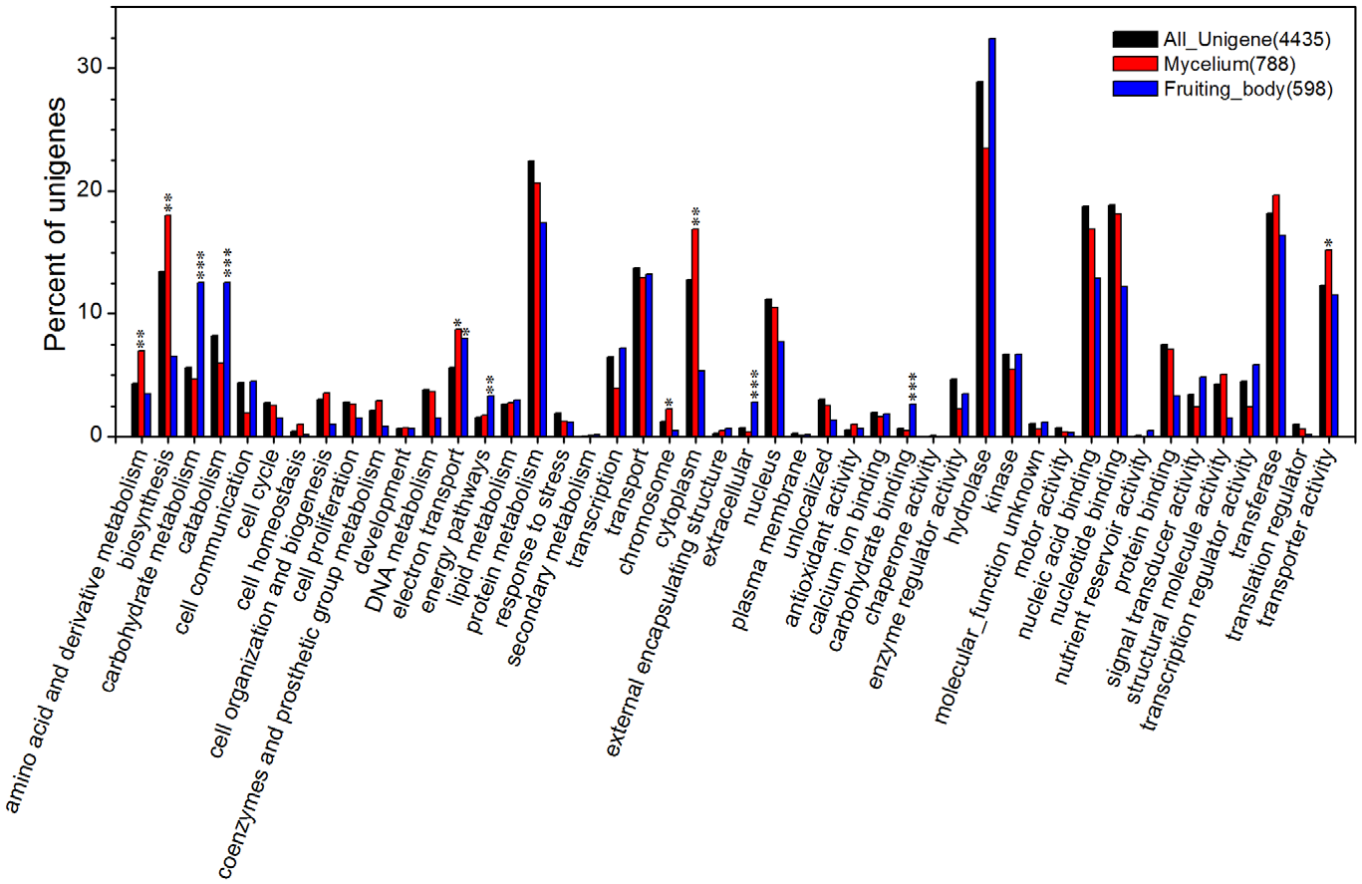
GO annotation of DEGs. The percentage of unigenes contained in a particular GO group is shown for all 4,435 present unigenes, 788 mycelium DEGs and 598 fruiting body DEGs and these are represented as in black, red, and blue, respectively. Asterisks indicate a significant overrepresentation of functional categories compared to the functional categories of 4,435 present genes (*p<0.05; **p<0.01; ***p<0.001). 6 GO terms were overrepresented in mycelium DEGs and 6 GO terms were enriched in the fruiting body DEGs.

From the functional annotation of the whole *G. lucidum* transcriptome, GO annotation of DEGs was extracted and subjected to GO functional enrichment analysis. While the number of DEGs in mycelium was less than that of the fruiting body, the number of unigenes with GO annotations in mycelium DEGs (788) was more than that of the fruiting body (598). As shown in Figure 6, the GO terms “amino acid and derivative metabolism” (P≤0.01), “biosynthesis” (P≤0.01), “electron transport” (P≤0.01), “chromosome” (P≤0.05), “cytoplasm” (P≤0.01) and “transporter activity” (P≤0.05) were significantly over-represented among the mycelium DEGs. In contrast, GO categories “carbohydrate metabolism” (P≤0.0001), “catabolism” (P≤0.001), “electron transport” (P≤0.05), “energy pathways” (P≤0.01), “extracellular” (P≤0.0001) and “carbohydrate binding” (P≤0.0001) were over-represented in fruiting body DEGs. These results indicated that biosynthesis activity (such as, “amino acid and derivative metabolism” and “biosynthesis”) was much higher at the transcriptional level in mycelium compared with the fruiting body and that the carbohydrate metabolism is essential in *G. lucidum* fruiting bodies. The GO categories: “protein metabolism”, “transport”, “hydrolase” and “transferase” were expressed at high levels in both mycelium and fruiting body, demonstrating that these metabolic activities are essential to both development stages.

Among the DEGs, the general annotation level of biosynthesis metabolism and degradation metabolism in both KEGG annotation and GO annotation were almost in accordance with each other in some aspects. For example, in KEGG annotation of DEGs, “ATP synthesis” and “TCA cycle” were up-expressed in mycelium whereas “starch and sucrose metabolism” and “N-glycan degradation” were up-regulated in fruiting body. Correspondingly, in GO annotation of DEGs, “biosynthesis” and “protein metabolism” were over-represented in mycelium whereas “carbohydrate metabolism” and “hydrolase” were over- represented in fruiting body. This indicated the completeness of the transcriptome and the accuracy of our analysis.

#### 3) The Ganoderic acids biosynthesis pathway

**Figure 7:**
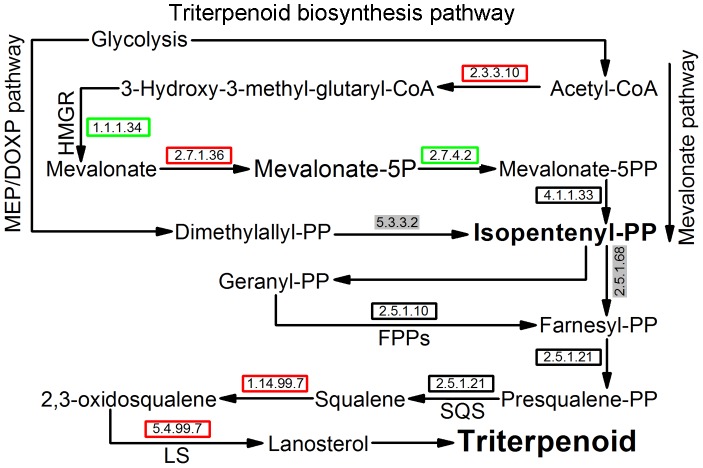
Metabolic pathway of Ganoderic acids. The expression levels of unigenes involved in this pathway were confirmed by transcriptome analysis and quantitative PCR. Red boxes represent pathways containing unigenes that show up-regulated expression in mycelium, green boxes represent up-regulated expression in the fruiting body. Black boxes represent pathways having common expression levels between mycelium and fruiting body. Words in a grey shadow represent that no unigenes were identified in this process after *G. lucidum* transcriptome analysis.

Ganoderic acids (GAs) are important pharmaceutical components in *G. lucidum*, which have been shown to have anti-tumor activity, roles in immunomodulation, hypoglycemic treatment and hepatoprotection [60]–[62]. To deeply understand GAs, the biosynthesis pathway of this ingredient was analyzed. From Figure 5, the metabolic pathway “Terpenoid backbone biosynthesis” in GA biosynthesis showed increased expression in mycelium at the overall transcriptional level and the metabolic processes of this pathway was elaborated by using the KEGG tool (Figure 7).

Two pathways, 2-methyl-D-erythritol–4-phosphate/1-deoxyxylulose-5-phosphate (MEP/DOXP) pathway and mevalonate pathway (MVP), are involved in the GA biosynthesis. The mevalonate pathway is the main GA biosynthesis pathway. The mevalonate pathway can be divided into four main processes (Figure 7). The first process involves the conversion of acetyl-coenzyme A to isopentenyl pyrophosphate (IPP) in which the 3-hydroxy-3-methyglutaryl coenzyme A reductase (HMGR) catalyzes the synthesis of mevalonate. Second, the action of various prenyltransferases generate high order terpenoid building blocks (geranyl pyrophosphate (GPP) and farnesyl pyrophosphate (FPP)) from the IPP precursor, which is also involved in the conversion of GPP to FPP by farnesyl diphosphate synthase (FPPs). Then these intermediates are converted into lanosterol by squalene synthase (SQS) and lanosterol synthase (LS). Finally, the lanosterol is metabolized into triterpenoid [63], [64].

Furthermore, unigenes involved in “Terpenoid backbone biosynthesis” pathway were selected out by KEGG annotation (Table 2). 13 unigenes were identified and shown to be involved in terpenoid backbone biosynthesis pathway, of which 7 unigenes showed up-regulated expression in mycelium and 6 unigenes did not show significant expression changes during the two development stages. These unigenes were matched to 9 known proteins by using BLASTx algorithm against the nr database with an E-value cutoff of 10^−10^. *Unigene6227_All*, *Unigene803_All, Unigene111_All* and *Unigene4718_All* showed high identity with *HMGR* [GeneBank: ABY84849], *FPPs* [GeneBank: ACB37020], *SQS* [GeneBank: ABF57214] and *LS* [GeneBank: ADD60469], respectively [65]–[68] (Figure S3). The other five proteins were: mevalonate kinase (*Unigene25033_All* and *Unigene14395_All*), phosphomevalonate kinase (*Unigene6822_All*), mevalonate disphosphate decarboxylase (*Unigene6187_All*), geranylgeranyl pyrophosphate synthetase (*Unigene688_All*) and squalene monooxygenase (*Unigene2082_All* and *Unigene5606_All*).

**Table 2:** *G. lucidum* unigenes putatively involved in the terpenoid backbone biosynthesis pathway.

ecNum	Kegg_ID	Gene_ID	Length(nt)	M_RPKM	F_RPKM	Log_2_(M_RPKM/F_RPKM)	Gene Description
2.3.3.10	K01641	Unigene27186_All	309	465.9	95.8	2.3	Hydroxymethylglutaryl-CoA synthase
		Unigene6833_All	1217	475.8	98.5	2.3	Hydroxymethylglutaryl-CoA synthase
1.1.1.34	K00021	Unigene6227_All	1252	234.9	185.4	0.3*	Hydroxymethylglutaryl-CoA synthase (HMGR)
2.7.1.36	K00869	Unigene25033_All	987	37.5	8.9	2.1	Mevalonate kinase
		Unigene14395_All	1144	30.9	13.6	1.2	Mevalonate kinase
2.7.4.2	K00938	Unigene6822_All	1304	49.3	69.6	−0.5*	Phosphomevalonate kinase
4.1.1.33	K01597	Unigene6187_All	1477	167.1	110.4	0.6*	Mevalonate disphosphate decarboxylase
2.5.1.10	K13789	Unigene803_All	1281	174.8	139.3	0.3*	Farnesyl pyrophosphate synthase (FPPS)
		Unigene688_All	1601	60.4	92.5	−0.6*	Geranylgeranyl pyrophosphate synthetase
2.5.1.21	K00801	Unigene111_All	3682	94.8	78.3	0.3*	Squalene synthase (SQS)
1.14.99.7	K00511	Unigene2082_All	414	143.5	30.7	2.2	Squalene monooxygenase
		Unigene5606_All	522	62.6	17.8	1.8	Squalene monooxygenase
5.4.99.7	K01852	Unigene4718_All	1890	47.7	17.5	1.5	Lanosterol synthase (LS)

The ecNum column represents a specific process of a metabolic pathway. The value of Log_2_(M_RPKM/F_RPKM) represents the differentially expressed status of unigenes during the two development stages. Positive values indicate increased expression in mycelium while negative values represent decreased expression in the fruiting body.

* Expression differences were considered to be not significant.

**Figure 8:**
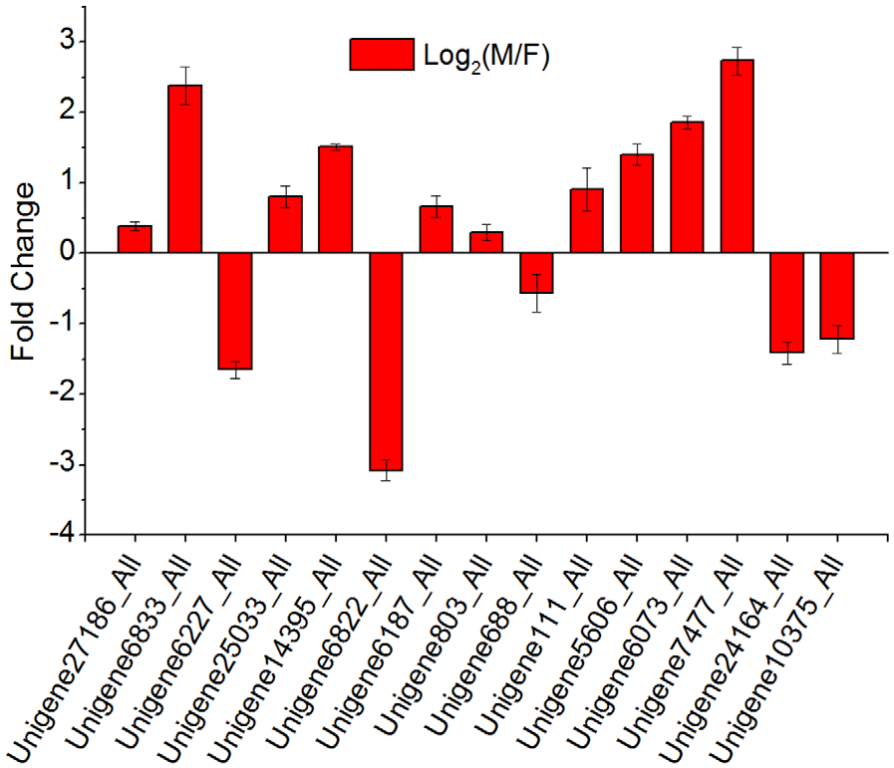
Quantitative analysis of unigenes from *G. lucidum* mycelium and fruiting body. Candidate genes from *G. lucidum* mycelium and fruiting body were analyzed quantitatively by qPCR. Fold changes are denoted as the log_2_ value of the ratio of gene expression in mycelium to expression in the fruiting body. The top 11 unigenes in the figure were from Table 2, which are involved in the GA biosynthesis pathway. *Unigene2082_All* and *Unigene4718_All* are involved in the GA pathway but were not detected in the fruiting body by qPCR. The last 4 unigenes were from Table 3 and *Unigene6029_All* was not detected in fruiting body. (Mean ± SD, N = 3).

The expression levels of these unigenes were confirmed by quantitative PCR (Figure 8). Our qPCR results are in accordance with the transcriptome data with the exception of *Unigene6227_All* (Table 2). *Unigene6227_All* was up-regulated in fruiting body while *Unigene2082_All* and *Unigene4718_All* were only detected in mycelium. Previous work had demonstrated that *HMGR* (*Unigene6227_All*), *FPPs (Unigene803_All), SQS (Unigene111_All) and LS (Unigene4718_All)* were highly expressed during the primordia stage [65]–[68], in contrast, our transcriptome data and qPCR results showed that *Unigene4718_All* was up expressed in mycelia. Previous reports have confirmed that the environmental factors can influence the ganoderic acid production by changes in oxygen [69], light [70], PH [71] and heavy metal ion [72]. So, it is comprehensible for the little discrepancy between our results and previous works.

According to KEGG annotation of DEGs and previous reports regarding fungal development [48], [73], [74], 5 uingenes were chosen for qPCR experiment. These genes showed high identity to ATP synthase F1 (*Unigene6073_All*), citrate synthase (*Unigene7477_All*), EXG1 (*Unigene24164_All*), hydrophobin 2 (*Unigene6029_All*) and cytochrome P450 (*Unigene10375_All*) (Table 3). With the exception of *Unigene6029_All*, which was only detected in mycelium, there was a strong correlation between our qPCR results and transcriptome data for these 5 unigenes (Figure 8).

**Table 3:** Orthologues of chosen unigenes according to the KEGG annotation of DEGs.

Unigene_ID	Unigene length	M_RPKM	F_RPKM	Log_2_(M_RPKM/F_RPKM)	Homologous gene product[Accession No.]	Organism	Identity(%)	E-value
Unigene6073_All	1836	862.88	227.93	1.92	ATP synthase F1 [XP_001831471]	*Coprinopsis cinerea*	89	0
Unigene7477_All	1689	574.20	110.70	2.38	Citrate synthase [BAF31127]	*Phanerochaete chrysosporium*	84	0
Unigene24164_All	977	3.04	76.20	−4.65	EXG1 [AAO32563]	*Lachancea kluyveri*	44	4E-24
Unigene6029_All	750	167.81	41.74	2.01	Hydrophobin 2 [AAF61066]	*Lentinula edodes*	61	2E-23
Unigene10375_All	596	15.86	407.38	−4.68	cytochrome P450 [BAK09378]	*Postia placenta*	64	2E-80

Genes for analysis were chosen on the basis of KEGG annotation of DEGs. The tBLASTn algorithm was used to search for similar unigenes in the *G. lucidum* transcriptome against the nr database.

The GAs biosynthesis pathway is complex and such environmental stimuli may affect the expressional level of some genes in this pathway. Further research is needed to further understand this complex metabolic pathway.

### Putative Wood-degrading Genes in *Ganoderma Lucidum*

The basidiomycetes: *Ganoderma lucidum*, *Laccaria bicolor*, *Coprinopsis cinerea*, *Schizophyllum commune*, *Phanerochaete chrysosporium* and *Postia placenta* all belong to the subphyla Agaricomycotina with the top five being white-rot fungi and the last one being brown-rot fungus [48]. Most white-rot fungi belong to the basidiomycete and can degrade components of plant cell walls such as cellulose, hemicellulose, and lignin [75], [76]. Lignin-degrading enzymes are comprised of lignin oxidases (LO families) and lignin-degrading auxiliary enzymes (LDA families) [8].

To identify related genes from *G. lucidum* transcriptome, BLASTx was performed against the fungal oxidative lignin enzymes (FOLymes) database (http://foly.esil.univ-mrs.fr/). *G. lucidum* has 13 and 9 potential members in LO family and LDA family, respectively (Table 4). The 13 putative genes of LO family consists of 10 laccase genes (LO1), one peroxidases gene (LO2) and two CDH genes (LO3). The LO1 (laccase) in *G. lucidum* have been extensively studied [6], [7], [77]. The 9 potential LDA enzymes consists of one aryl-alcohol oxidase (LDA1), 5 glyoxal oxidases (LDA3), one benzoquinone reductase (LDA7) and two alcohol oxidases (LDA8).

**Table 4:** BLASTx results of the *G. lucidum* transcriptome against FOLymes and CAZymes database.

	FOLymes												CAZymes				
	LO1	LO2	LO3	LDA1	LDA2	LDA3	LDA4	LDA5	LDA6	LDA7	LDA8	ΣFOLymes	GH	GT	PL	CE	ΣCAZymes
Total numbers ofmatched unigenes	10	1	2	1	0	5	0	0	0	1	2	22	78	40	0	2	120
Up-expressed inmycelium	0	1	0	0	0	3	0	0	0	0	0	4	7	12	0	0	19
Up-expressed infruiting body	0	0	0	0	0	1	0	0	0	1	2	4	32	9	0	1	42

LO1, laccases; LO2, peroxidases; LO3, cellobiose dehydrogenases; LDA1, aryl alcohol oxidases; LDA2, vanillyl-alcohol oxidases; LDA3, glyoxal oxidases; LDA4, pyranose oxidases; LDA5, galactose oxidases; LDA6, glucose oxidases; LDA7, benzoquinone reductases; LDA8, alcohol oxidases; GH, glycoside hydrolases; GT, glycosyl transferases; PL, polysaccharide lyases; CE, carbohydrate esterases. Analysis was performed by using the BLASTx program with an E-value cutoff of 10^−20^ and an identity threshold of 60%.

Enzymes involved in carbohydrate metabolism include: Carbohydrate esterases (CE), Glycoside hydrolases (GH), Glycosyl transferases (GT), and Polysaccharide lyases (PL), which are commonly referred to as carbohydrate-active enzymes (CAZymes, http://www.cazy.org/) [78], [79]. Our BLASTx results showed that 120 unigenes from *G. lucidum* transcriptome had high identity to CAZymes. Out of these unigenes, 78 homologs belonged to the GH superfamily, 40 candidates to glycosyl transferases and 2 candidates to carbohydrate esterases. In the GH superfamily, GH5 possessed 12 unigenes and was the most dominant, followed by GH31 (11 unigenes), GH3 (7 unigenes) and GH16 (7 unigenes). Information of unigene distribution in the GH superfamily is listed in Table S5.

The differential expression of these putative lignocellulose degrading genes between *G. lucidum* mycelium and fruiting body is elaborated in Table 4. In summary, the expression levels of FOLymes between the two developmental stages did not reveal any significant difference, whereas CAZymes, especially the GH superfamily, were found to be generally up-regulated in the fruiting body.

### Conclusions

*G. lucidum* has important herbal medicinal value in Asia. While the function of many its pharmaceutical components (e.g. ganoderic acids, polysaccharides, *LZ-8*, etc.) has been extensively studied [10], [25], [61], little genomic information remains available on this fungus.

In the current work, a transcriptome database of *G. lucidum* was constructed. From the two different developmental stages (mycelium and fruiting body), we identified differentially expressed genes by transcriptome analysis and found that numerous metabolic pathways relating to biosynthesis were significantly over-expressed at the transcriptional level during the mycelium stage, especially in the biosynthesis of important pharmaceutical components in *G. lucidum* such as ganoderic acids. During the fruiting body stage, unigenes involved in degradation activity were highly expressed such as the glycan degradation. The biosynthesis pathway of GA is complex and further analytical work is required. While *G. lucidum* is an important medicinal fungus in Asia, little developmental information is available at present, our transcriptome provides a resource for further extensive studies on developmental processes in this important fungi.

Here, we show that transcriptome sequencing is an effective technology for analyzing complex functions of fungal genomes. To our knowledge, this is the first publication of the *G. lucidum* transcriptome using Illumina sequencing technology.

## Materials and Methods

### Sample Preparation

Mycelia were cultured from a central *Ganoderma lucidum* source (mycelia discs, r = 3 mm) on a potato dextrose agar (PDA) medium at 20°C in the dark with 90% air humidity. After 16 days, the mycelia totally covered the petri dish and 1.5 g of mycelia were collected [12]. The mycelia were then transferred to basswood medium and stored in light at 28°C, 90% air humidity until fruiting-bodies were formed [57]. After nine weeks, 1.5 g of fruiting bodies were collected. Samples from the two developmental stages were frozen at −80°C until RNA extraction.

### cDNA Library Construction and Sequencing

Total RNA from *Ganoderma lucidum* mycelium and fruiting body was extracted using TriZol reagent (Promega) according to the manufacturer’s protocol. RNA quantity and quality were checked by a NanoDrop 1000 spectrophotometer (NanoDrop Technologies) and an Agilent 2100 Bioanalyzer (Agilent Technologies, Santa Clara, CA) and the two samples had RNA Integrity Number (RIN) values greater than 8.5. The mRNA was concentrated from equal amounts of total RNA (20 µg per sample) using oligo (dT) magnetic beads. All mRNA was broken into short fragments (200–700 nt) by adding fragmentation buffer. These short sequences were used for the first strand cDNA synthesis using reverse transcriptase and random primers followed by the second strand cDNA synthesis with DNA polymerase I and RNaseH.

The cDNA fragments were purified using a QiaQuick PCR extraction kit. These purified fragments then were washed with EB buffer for end reparation poly (A) addition and ligated to sequencing adapters. Following agarose gel electrophoresis and extraction of cDNA from gels, the cDNA fragments with the lengths of 200 bp (±25 bp) were purified and enriched by PCR to construct the final cDNA library.

Two cDNA libraries (*G. lucidum* mycelium and fruiting body) were sequenced at Beijing Genome Institute (BGI, Shenzhen, China) on the Illumina sequencing platform (Illumina HiSeq™ 2000) using paired-end technology in a single run. The original images process to sequences, base-calling and quality value calculation were performed by the Illumina GA Pipeline (version 1.6), in which 90 bp paired-end reads were obtained.

### De novo Assembly and Analysis of Transcriptome

Before assembly, the 90-bp raw reads were filtered to obtain the high-quality clean reads by removing adaptor sequences, the reads containing more than 10% “N” rate (the “N” character representing ambiguous bases in reads) and low quality sequences (reads in which more than 50% of the bases had a quality value ≤10).

*De novo* assembly of the clean reads was performed using SOAPdenovo program (http://soap.genomics.org.cn) which implements a de Bruijn graph algorithm [80]. Briefly, the overlap information from the clean reads was used to construct high-coverage contigs without N. Then the reads were realigned onto contigs. To connect the contigs, N was applied to represent unknown sequences between each pair of contigs, and a scaffold was built. Paired-end reads were used again for gap filling between scaffolds to obtain unigenes which have the least ‘N’s and cannot be extended at either end. Assembled unigenes from each sample were further processed by using sequence clustering software, TGI Clustering tools and Phrap.

The BLASTx algorithm was used to search for homologous sequences against the NCBI nr database and LCSPP database using an E-value cutoff of 10^−5^. The LCSPP database contained some fungal genomes which were released by the U.S. Department of Energy Joint Genome Institute (http://www.jgi.doe.gov/). Functional annotation of these unigenes was determined by using the BLASTx program against the LCSPP database, followed by additional manipulation with perl scripts.

### Analysis of Differentially Expressed Genes (DEGs)

To identify the differentially expressed genes between the two developmental stages, the false discovery rate (FDR) method was used to determine the threshold of P-value in multiple tests [81], [82]. A FDR ≤0.001 and an absolute value of the log2 ratio ≥1 were used as threshold to determine significant differences in gene expression between the two developmental stages.

Functional annotation was applied to obtain enriched genetic annotation from mycelium and fruiting body. The p-values for significant overpresentation of a particular GO category were calculated by using the hypergeometric distribution.

### Quantitative Real time PCR

Total RNA was extracted from mycelium and fruiting body of *G. lucidum* using TRIzol® by following the manufacturer’s protocol. Total RNA was resuspended in 100 µl of nuclease-free water and the concentration was measured (1.5 µg/µl, respectively) by using a NanoVue (GE Healthcare NanoVue). About 2 µg of total RNA was used as template to synthesize first-strand cDNA by using a M-MLV Reverse Transcriptase kit (Promega). The resultant cDNA was stored at −20°C until further use.

Unigenes of interest were subjected to quantitative PCR (qPCR) analysis. Primers were designed on the basis of matched parts against known genes in the KEGG database with our unigenes by using Primer 3. Detailed information of these primers is shown in Table S6. Amplifications were performed using 0.5 µl (10 µM) of the specific primers, 10 µl of SYBR® qPCR Mix (TOYOBO) and 40 ng of cDNA in a final volume of 20 µl. The cycling parameters were 95°C for 4 min followed by 45 cycles of 95°C for 10 s, 57°C for 15 s and 72°C for 20 s. 3 replicates were performed for each gene tested in real time PCR reactions. The *G. lucidum* glyceraldehyde-3-phosphate dehydrogenase (*Gl-GPD*) gene (*Unigene27520_All*, 100% similarity) was used as the internal reference gene [66], [83]. Relative gene expression was analyzed by the 2^−ΔΔCT^ method.

### CAZy and FOLy Annotation

Unigenes from *Ganoderma lucidum* transcriptome that are related to carbohydrate-related enzymes and ligin oxidative enzymes were confirmed by BLASTx searches against CAZy and FOLy database using a threshold of 1.0E^−20^. Unigenes possessing a sequence identity greater than 60% with these biochemically characterized enzymes were considered to be a CAZyme or FOLyme.

### Data Deposition

The raw Illumina sequencing dataset of *Ganoderma lucidum* was submitted to the NCBI Sequence Read Archive under the accession number of SRA036392.

## Supporting Information

Figure S1 **BLASTx result distribution of** ***G. lucidum*** **transcriptome against the LCSPP database.** All unigenes from the *G. lucidum* transcriptome were aligned against the LCSPP database using BLASTx with a cutoff E-value of 1.0E^−5^. The percentages represent the ratio between the BLASTx result against one of the five fungal genomes and all unigenes. 22.08% of 28,210 unigenes showed high identity to *Postia placenta*, followed by *Phanerochaete chrysosporium* (21.36%), *Schizophyllum commune* (16.73%), *Coprinopsis cinerea* (11.87%) and *Laccaria bicolor* (10.25%).
(TIF)

Figure S2 **Histogram presentation of clusters of COG categorization.** Out of 28,210 unigenes, 5,217 sequences have a COG classification among the 25 categories.
(TIF)

Figure S3 **Sequence alignment of** ***HMGR*** **,** ***FPPs*** **,** ***SQS*** **and** ***LS*** **against the** ***Ganoderma lucidum*** **transcriptome.** (A) Sequence alignment of the *HMGR* gene [GeneBank: ABY84849] with *Unigene6227_All* produced 99% identity. (B) Alignment of the *FPP* gene [GeneBank: ACB37020] produced 97% identity to *Unigene803_All*. (C) Sequence alignment of the *SQS* gene [GeneBank: ABF57214] produced an identity of 95% with *Unigene111_All*. (D) *LS* [GeneBank: ADD60469] has 97% identity to *Unigene4718_All*. All alignments were performed using tBLASTn program, ClustalX 2.0.11 and Jalview 2.6.1 software.
(TIF)

Table S1 **Unigenes annotated by GO.** 4,435 unigenes from the *G. lucidum* transcriptome were annotated against the LCSPP database.
(XLS)

Table S2 **Unigenes annotated by KEGG.** From the transcriptome, 1,396 unigenes were annotated using the LCSPP database and were classified into 112 categories. These pathways were further grouped into 16 broader metabolic pathway classes.
(XLS)

Table S3 **Annotation of the** ***G. lucidum*** **transcriptome by InterPro.** 4,619 unigenes from the *G. lucidum* transcriptome resulted in InterPro annotations of 2,612 categories. Most of the annotated unigenes were found to match to more than one conserved protein domain.
(XLS)

Table S4 **Differentially expressed genes (DEGs) between** ***G. lucidum*** **mycelium and fruiting body.** From the *G. lucidum* mycelium and fruiting body, 8,330 unigenes were DEGs, of which 446 unigenes showed specific expression patterns in mycelium and 913 unigenes were specific in the fruiting body.
(XLS)

Table S5 **Classification of putative GH genes from the** ***G. lucidum*** **transcriptome.**
(XLS)

Table S6 **Primers used in the qPCR of** ***G. lucidum*** **unigenes.**
(XLS)
