# Supplementary figures and images for: Deep Insight into the Ganoderma lucidum by Comprehensive Analysis of Its Transcriptome
Source: PLoS One. 2012 Aug 27;7(8):e44031. doi: 10.1371/journal.pone.0044031 (PMC3428325; doi:10.1371/journal.pone.0044031)

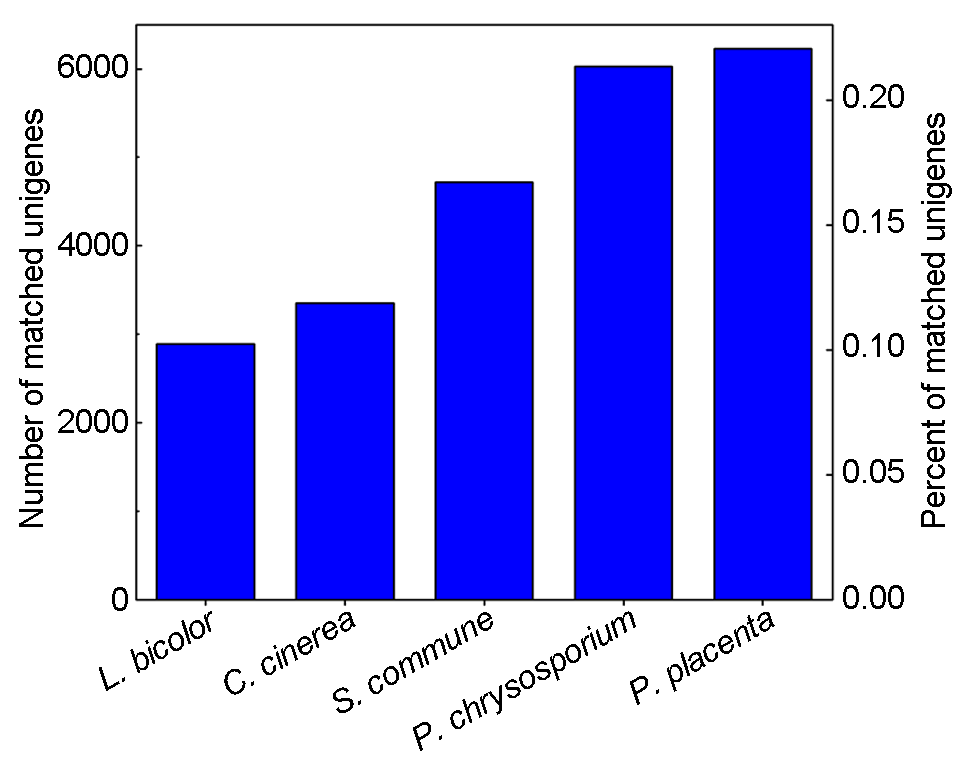

Supplement: Figure S1 — BLASTx result distribution of G. lucidum transcriptome against the LCSPP database. All unigenes from the G. lucidum transcriptome were aligned against the LCSPP database using BLASTx with a cutoff E-value of 1.0E−5. The percentages represent the ratio between the BLASTx result against one of the five fungal genomes and all unigenes. 22.08% of 28,210 unigenes showed high identity to Postia placenta, followed by Phanerochaete chrysosporium (21.36%), Schizophyllum commune (16.73%), Coprinopsis cinerea (11.87%) and Laccaria bicolor (10.25%). (TIF) [file pone.0044031.s001.tif]

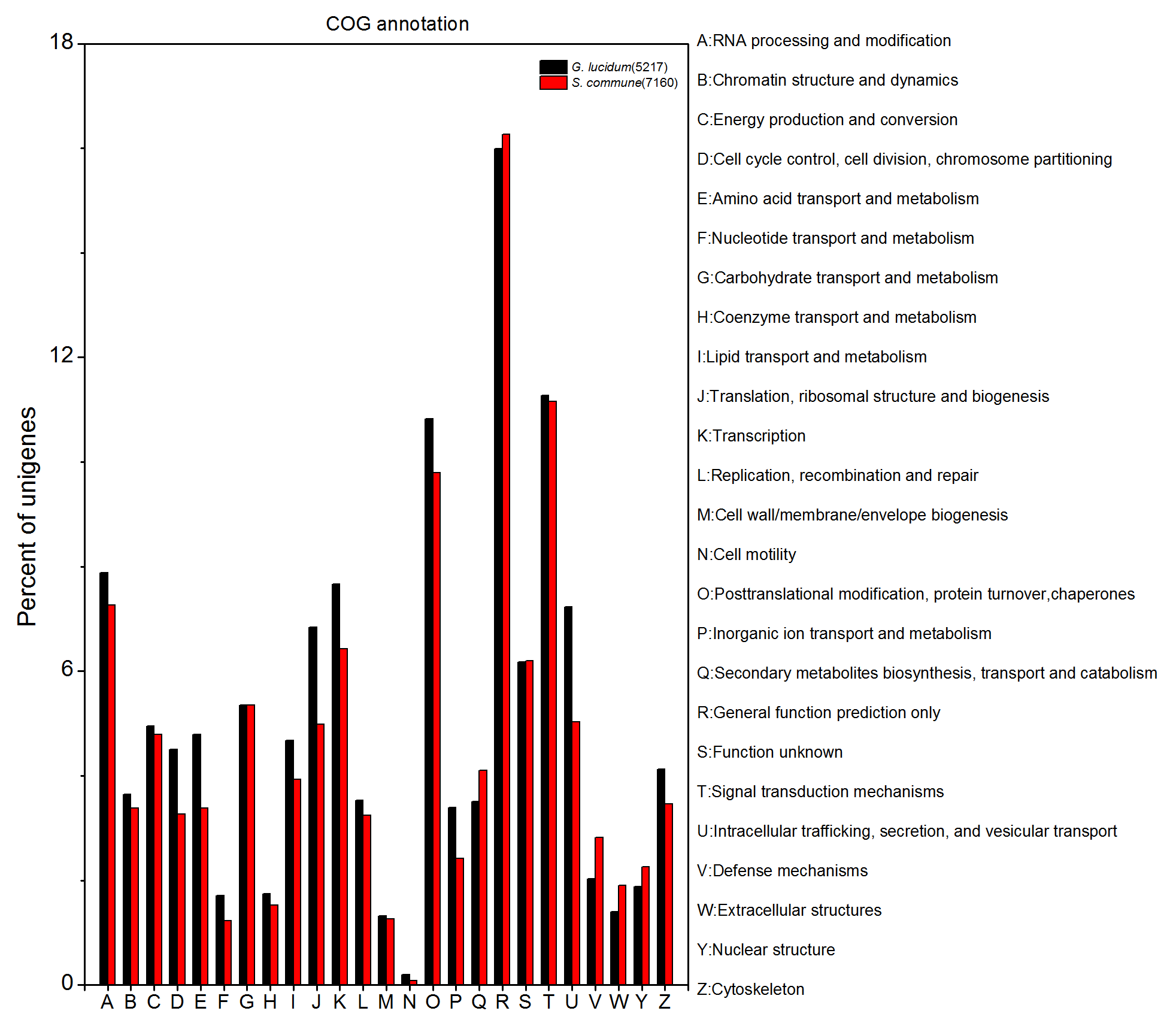

Supplement: Figure S2 — Histogram presentation of clusters of COG categorization. Out of 28,210 unigenes, 5,217 sequences have a COG classification among the 25 categories. (TIF) [file pone.0044031.s002.tif]

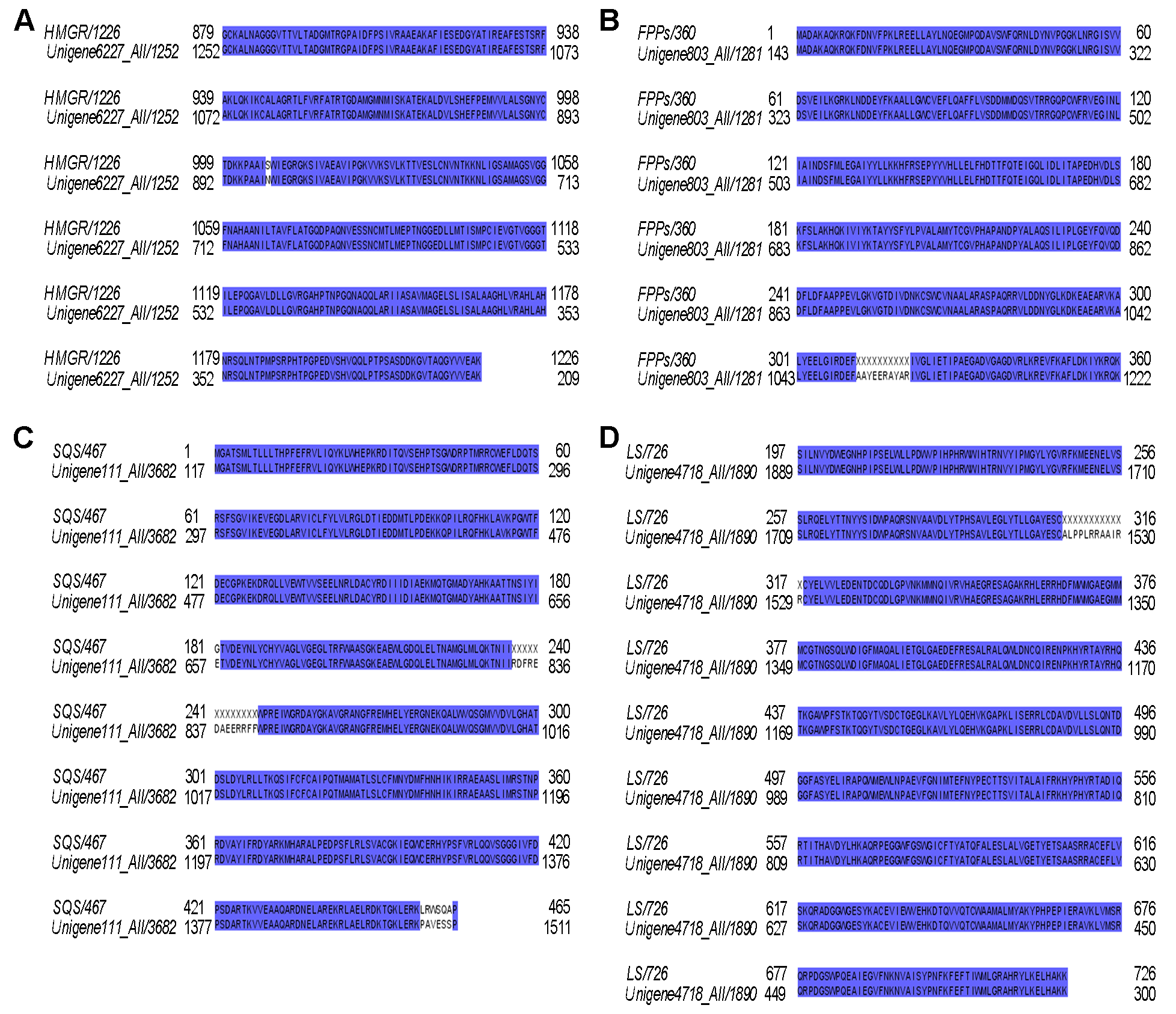

Supplement: Figure S3 — Sequence alignment of HMGR , FPPs , SQS and LS against the Ganoderma lucidum transcriptome. (A) Sequence alignment of the HMGR gene [GeneBank: ABY84849] with Unigene6227_All produced 99% identity. (B) Alignment of the FPP gene [GeneBank: ACB37020] produced 97% identity to Unigene803_All. (C) Sequence alignment of the SQS gene [GeneBank: ABF57214] produced an identity of 95% with Unigene111_All. (D) LS [GeneBank: ADD60469] has 97% identity to Unigene4718_All. All alignments were performed using tBLASTn program, ClustalX 2.0.11 and Jalview 2.6.1 software. (TIF) [file pone.0044031.s003.tif]
